# *In Vivo* Studies on the Influence of Bacteriophage Preparations on the Autoimmune Inflammatory Process

**DOI:** 10.1155/2017/3612015

**Published:** 2017-10-22

**Authors:** Ryszard Międzybrodzki, Jan Borysowski, Marlena Kłak, Ewa Jończyk-Matysiak, Bożena Obmińska-Mrukowicz, Agnieszka Suszko-Pawłowska, Barbara Bubak, Beata Weber-Dąbrowska, Andrzej Górski

**Affiliations:** ^1^Bacteriophage Laboratory, Hirszfeld Institute of Immunology and Experimental Therapy, Polish Academy of Sciences, 53-114 Wroclaw, Poland; ^2^Phage Therapy Unit, Hirszfeld Institute of Immunology and Experimental Therapy, Polish Academy of Sciences, 53-114 Wroclaw, Poland; ^3^Department of Clinical Immunology, Transplantation Institute, Medical University of Warsaw, 02-006 Warsaw, Poland; ^4^Research and Development Center, Regional Specialized Hospital, 51-124 Wroclaw, Poland; ^5^Department of Biochemistry, Pharmacology and Toxicology, Faculty of Veterinary Medicine, Wroclaw University of Environmental and Life Sciences, 50-375 Wroclaw, Poland

## Abstract

Phage preparations used for phage therapy may have not only direct antibacterial action but also immunomodulating effects mediated by phages themselves as well as by bacterial antigens. Therefore phage application in patients with immune disorders, and especially with autoimmune diseases, requires special attention. The aim of this study was to investigate the effect of phage lysates (staphylococcal phages A3/R, phi200, and MS-1 cocktail, enterococcal phage 15/P,* Pseudomonas* phage 119x, and* E. coli* T4 phage) as well as purified T4 phage on the course of murine collagen-induced arthritis (CIA), commonly used as an animal model of rheumatoid arthritis. Intraperitoneal application of phage lysates or purified T4 phage did not aggravate the course of autoimmune joint disease. Moreover, although endotoxins are known to potentiate CIA, the systemic administration of phage lysate of* Pseudomonas aeruginosa*, which contains debris of this Gram-negative bacillus, did not significantly influence CIA although the sonicate of the corresponding bacterial strain did. Interestingly, a purified T4 phage revealed some anti-inflammatory activity when applied under the therapeutic scheme. Our preliminary results do not suggest that phages may aggravate the symptoms of rheumatoid arthritis. In contrast T4 phage may even exert an immunosuppressive effect.

## 1. Introduction

The threat of emerging infections caused by antibiotic resistant bacteria results in growing interest in bacteriophage therapy, which is considered as a potential alternative to antibiotics [1]. A number of controlled clinical studies have been conducted and others are underway [2, 3]. So far phage therapy has been used extensively in Georgia as a standard treatment as well as to a lesser degree in Poland under the rules of therapeutic experiments [4]. Phages and their preparations (purified and/or so-called phage lysates) which are used for phage therapy may not only have direct antibacterial action but can also have immunomodulatory activities in the human host [5]. Although bacteriophages are generally considered to be nonpathogenic for humans, they can elicit some immunomodulatory effects including antibody production, induction of cytokines (such as interferon), alterations in T-cell mediated immunity, or a decrease in free radicals production by endotoxin activated neutrophils. Staphylococcal phage lysates were registered and applied in the last century as immunostimulators in humans (e.g., SPL, Staphage Lysate, produced by Delmont Laboratories, which is now registered only for veterinary use in dogs for the treatment of canine pyoderma) [5, 6]. Therefore phage application in patients with immune disorders and especially with autoimmune diseases, which are caused by a failure in the tolerance to self-antigens, requires special attention.

Our aim was to investigate the effect of phages on the course of autoimmune disease. For the study we chose murine collagen-induced arthritis (CIA), which is commonly used as an animal model of rheumatoid arthritis, one of the most prevalent autoimmune diseases in humans, due to their pathophysiological similarities [7]. We evaluated the effect of standard* E. coli* T4 phage as well as our four therapeutic phages (staphylococcal, enterococcal, and* Pseudomonas*) and staphylococcal phage cocktail, which were among the most frequently used phages for experimental phage therapy conducted in the Phage Therapy Unit of the Hirszfeld Institute of Immunology and Experimental Therapy, Polish Academy of Sciences (HIIET PAS) in Wroclaw, Poland.

## 2. Materials and Methods

### 2.1. Animals

Experiments were carried out on the genetically CIA-susceptible DBA/1 LacJ male and female mice (The Jackson Laboratory, USA), 8–16 weeks old, housed under standard conditions with food and water ad libitum. All experiments were approved by the II Local Ethics Committee in Wroclaw, Poland (approval number 40/03, 19/2009, 16/2011, 36/2012, and 1/2014).

### 2.2. Phages

T4 phage was purchased from the American Type Culture Collection (ATTC, Rockville, Maryland, USA). Other phages used in this study were obtained from the HIIET PAS therapeutic bacteriophage collection.

The purified preparation containing* E. coli* T4 phage with a low endotoxin level (less than 10 EU/ml per 10^9^ of T4 pfu/ml) was prepared according to the method of Boratynski et al. consisting of ultrafiltration and chromatographic techniques, as described in detail elsewhere [8, 9]. Crude phage lysates were prepared according to the modified method of Ślopek et al. [10]. Briefly, phages and fresh culture (3–5 h, 37°C) of the host bacteria were incubated at 37°C in broth culture (peptone water) until complete lysis occurred (3–6 hours). Then the suspension was stored overnight at 4°C and next it was filtered through a 0.22-*μ*m Millipore filter. Phage lysates containing monovalent staphylococcal phage (A3/R, phi200), enterococcal phage (15/P), and* Pseudomonas* phage (119x) and* E. coli* T4 phage were prepared at the Bacteriophage Laboratory of the Institute of Immunology and Experimental Therapy, PAS in Wroclaw. An equal mixture of staphylococcal phages A5/80, P4/6409, and 676/Z (marked MS-1) was prepared at the Institute of Biotechnology, Sera and Vaccines BIOMED S.A. in Cracow. The phage titers were assessed using the double-layer agar method according to Adams [11].

### 2.3. Sonicates

Bacterial sonicates were obtained by complete sonication of a bacterial suspension used for phage propagation.* S. aureus* 19930 (host for A3/R phage),* S. aureus* 80 (host for A5/80 phage),* S. aureus* 11788 (host for 676/Z phage),* S. aureus* 6409 (host for phi200 and P4/6409 phages),* E. faecalis* 26589 (host for 15/P phage), and* P. aeruginosa* 119x (host for 119x phage) were from the Polish Collection of Microorganisms (HIIET PAS, Poland), whereas* E. coli* B strain (host for T4 phage) was from the ATTC (Rockville, Maryland, USA).

### 2.4. Immunization with Collagen

Antigen preparation for induction of arthritis was done according to the protocol described by Rosloniec et al. [12]. Collagen type II was dissolved at 4.0 mg/ml in 0.05 M acetic acid overnight at 4°C. Complete Freund's adjuvant was prepared by mixing heat-inactivated* Mycobacterium tuberculosis* (4.0 mg/ml; Difco) with incomplete Freund's adjuvant (Sigma). Antigen emulsion was prepared by mixing the dissolved collagen and adjuvant at a 1 : 1 ratio using two connected syringes at 4°C. CIA was induced according to Wooley [13]. The mice were immunized by intradermal injection of 0.05 ml of the emulsion, approximately 1-2 cm from the base of the tail (day 0). On day 21, they received the same booster dose of collagen emulsified in incomplete Freund's adjuvant (without* Mycobacterium tuberculosis*).

### 2.5. Treatment Protocol and Evaluation of CIA Severity

Mice were distributed randomly among groups. Treatment was started on day 22. The animals were administered intraperitoneally with a daily dose of 0.5 ml of purified phage, phage lysate, appropriate bacterial sonicate, or broth. The control group to phage lysate did not receive any injections, and the control group to purified phage received 0.5 ml of phosphate-buffered saline (PBS). Methotrexate (Sigma) dissolved in PBS was used as a positive control and administered in a dose of 10 mg/kg of body weight three times a week (every 2-3 days) from day 22 until day 42. As prophylactic application the preparations were applied from day –2 until day 5 or from day 0 until day 21. As therapeutic treatment the preparations were applied from day 22 until day 42.

**Figure 1:**
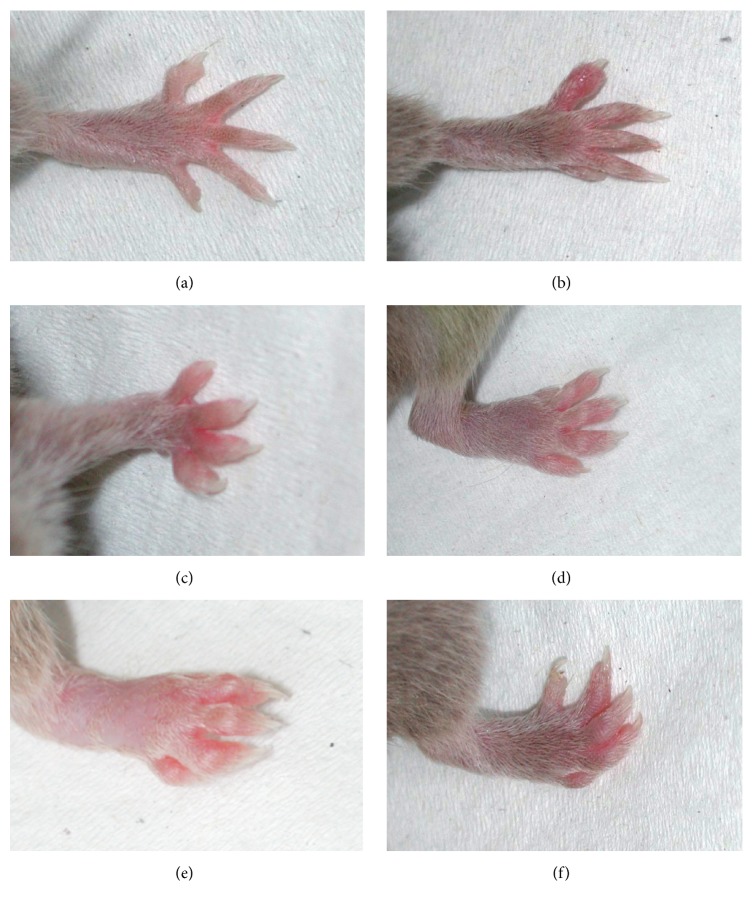
Illustration of intensity of arthritis in mice. (a) Normal front paw (CIA score: 0). (b) Edema of a single digit of the front paw (CIA score: 1). (c) Edema of four digits of the front paw (CIA score: 2). (d) Mild edema of an ankle and edema of all digits of the rear paw (CIA score: 3). (e) Intensive edema of an ankle and edema of all digits of the rear paw (CIA score: 4). (f) Edema and distortion of digits of the rear paw (CIA score: 4).

From day 21 the mice were visually examined every 3-4 days for signs of joint inflammation up to day 56. The method of assessment of degree of arthritis was evaluated using a five-degree score (as presented by Rosloniec et al.) modified by us (R. Międzybrodzki) to consider the different combinations of inflamed groups of joints and to reduce the influence of the subjectivity on the investigator's evaluation [12]. According to the detailed description in Table 1, points were granted for each toe and wrist/ankle, their sum for each paw was calculated, and it was converted into a degree of CIA. The CIA severity for a single animal was expressed as a sum of degree points of four paws (maximum: 16 degrees). Representative clinical images and scores are presented in Figure 1.

### 2.6. Tests of the Influence of MS-1 Preparation for Selected Parameters of the Immune Organs

#### 2.6.1. Organ Collection and Cell Isolation

Ten mice, randomly selected from each study group, were killed on day 63 by dislocation of cervical vertebra under halothane (Narkotan, Zentiva, Prague, Czech Republic) anesthesia and weighed. Their thymuses, spleens, and mesenteric lymph nodes were collected, weighed, and cooled to 4°C in PBS and further processed according to Suszko and Obmińska-Mrukowicz [14]. They were passed through nylon mesh and centrifuged at 2250 ×g (15 min, 4°C) at a 1 : 3 ratio in a density gradient of 1.076 (mixture of Ficoll-400 from Pharmacia Fine Chemicals and Urografin 76% from Bayer Schering Pharma). The cells were collected from the interphase, suspended in PBS with 1% bovine serum albumin (BSA, Sigma), and washed twice in this solution (375 ×g, 8 min, 4°C). Spleen resident erythrocytes were eliminated using a 0.84% solution of ammonium chloride (5 min of incubation at 37°C). Finally the total number of isolated cells was calculated and they were suspended in PBS with 1% BSA at a density of 1 × 10^7^ cells/ml for further analyses. The viability of each cell suspension was 90–98% according to a trypan blue dye-exclusion assay.

#### 2.6.2. Determination of Lymphocyte Populations

To assess the percentage of cell subpopulations, cell suspensions were incubated in darkness for 30 min at 4°C with appropriate fluorescein isothiocyanate (FITC) or R-phycoerythrin (RPE) conjugated monoclonal antibodies (rat anti-mouse CD4:FITC/CD8:RPE clone YTS191.1/KT15 or rat anti-mouse CD19:FITC/CD3:RPE clone 6D5/KT3, both from Serotec, Kidlington, UK) according to the manufacturer's protocol [14]. Then they were washed 3 times in PBS and the cell subpopulations were determined in a fluorescence flow cytometer (FACSCalibur, Becton Dickinson Biosciences, San Jose, CA, USA) and analyzed using CellQuest version 3.1.f software as previously described in detail [14].

### 2.7. Statistical Analysis

Results are presented as mean values (± standard error of the mean, SE) of the analyzed variable. To avoid overlapping and causing confusion as their actual value only upper or lower error bars representing SE (which were symmetric in all cases) are shown in figures. Mean arthritis scores (on each given examination day) in the study group were compared to the control group using the nonparametric Mann–Whitney* U* test or* t*-test when both variables were normally distributed. Differences in the weight, cell number, and percentage of cell populations of the thymus, spleen, and mesenteric lymph nodes were compared using one-way ANOVA followed by Scheffe's test. The differences between means were considered statistically significant when *p* < 0.05.

## 3. Results

**Figure 2:**
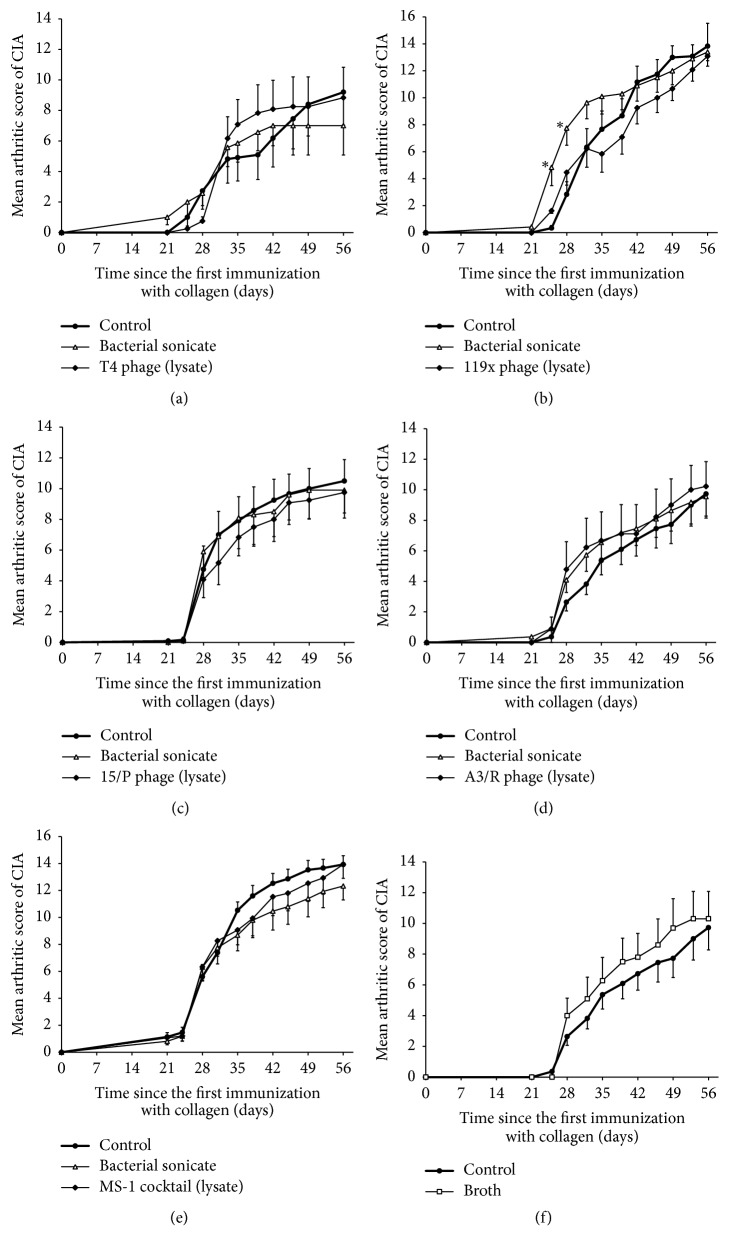
Influence of “therapeutic” intraperitoneal application (once daily from day 22 until day 42 after the first dose of collagen) of phage lysates, sonicate of their hosts, and broth (0.5 ml/mouse) on the course of CIA. (a) T4 phage lysate (2.5 × 10^9^ pfu/mouse) and its host* E. coli* B strain sonicate (number of mice in groups: *n* = 7–12). (b)* Pseudomonas* 119x phage lysate (6 × 10^5^ pfu/mouse) and its host* P. aeruginosa* 119x strain sonicate (number of mice in groups: *n* = 10–12). (c) Enterococcal 15/P phage lysate (3.25 × 10^6^ pfu/mouse) and its host* E. faecalis* 26589 strain sonicate (number of mice in groups: *n* = 10–12). (d) Staphylococcal A3/R phage lysate (4 × 10^8^ pfu/mouse) and its host* S. aureus* 19930 strain sonicate (number of mice in groups: *n* = 9–11). (e) MS-1 staphylococcal phage lysate containing an equal mixture of A5/80, P4/6409, and 676/Z phages, and sonicate of relevant mixture of their host strains (*S. aureus* 80,* S. aureus* 6409, and* S. aureus* 11788) (number of mice in groups: *n* = 15). (f) Broth used in the process of phage propagation (number of mice in groups: *n* = 10-11). ^*∗*^*p* < 0.05 in comparison to the control group (Mann–Whitney* U* test). For clarity reasons only upper or lower error bars representing SE are shown.

**Figure 3:**
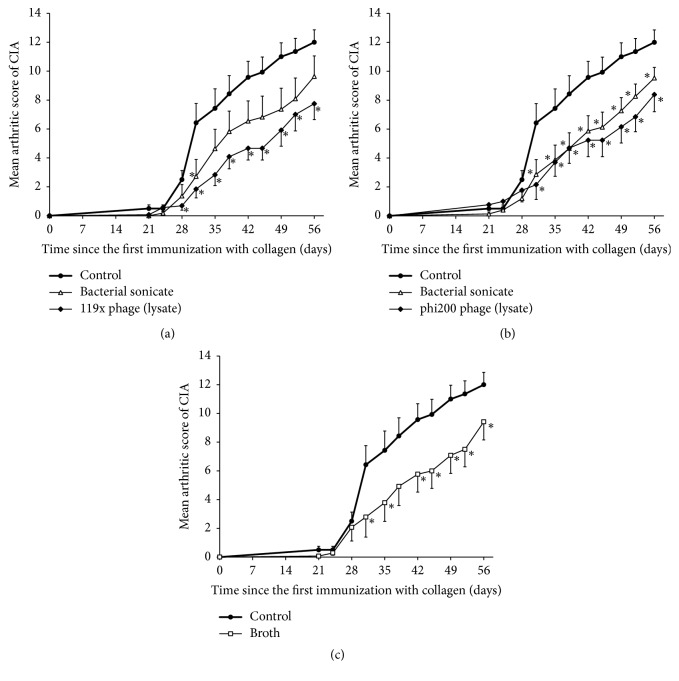
Influence of “prophylactic” intraperitoneal application (once daily from day 0 until day 21 after the first dose of collagen) of phage lysate, sonicate of their hosts, and broth (0.5 ml/mouse) given on the course of CIA. (a) 119x phage lysate (6 × 10^5^ pfu/mouse) and its host* P. aeruginosa* 119x strain sonicate (number of mice in groups: *n* = 12–15). (b) Phi200 phage lysate (1.4 × 10^7^ pfu/mouse) and its host* S. aureus* 6409 strain sonicate (number of mice in groups: *n* = 13–15). (c) Broth used in the process of phage propagation (number of mice in groups: *n* = 13-14). ^*∗*^*p* < 0.05 in comparison to the control group (Mann–Whitney* U* test). For clarity reasons only upper or lower error bars representing SE are shown.

The first signs of CIA appeared usually 28 days after the first immunization. The mean arthritic scores in the control group observed during experiments were the highest at day 56 and they were mainly between 7.3 and 13.9. This enabled us to observe both stimulatory and inhibitory effects of the studied agents. None of the studied monovalent phage lysates (T4, 119x, 15/P, A3/R) or staphylococcal phage cocktail MS-1 administered therapeutically (after the booster dose of collagen) showed a significant influence on the clinical course of CIA (Figures 2(a)–2(f)) during its application or two weeks after its termination (at day 56 in all cases the mean values of the arthritic score were very similar for phage lysates and control groups). The same result was observed for broth used for phage propagation and appropriate bacterial sonicates (Figures 2(a), 2(c)–2(f)) except sonicate of* P. aeruginosa* 119x (Figure 2(b)), a host for 119x phage, which significantly increased intensity of arthritis during the first week of its therapeutic application. Interestingly, extended prophylactic application of the* Pseudomonas* 119x and staphylococcal phi200 phage lysates from day 0 until day 21 caused significant inhibition of CIA induction from almost the beginning of the clinical observation for both preparations (Figures 3(a) and 3(b)). However, this phenomenon was attributed not only to the phage lysates but also to the broth and sonicate of* S. aureus* 6409, a host for phi200 phage (for sonicate of* P. aeruginosa* 119x strain CIA induction was also lowered but did not reach statistical significance) (Figures 3(a)–3(c)).

**Figure 4:**
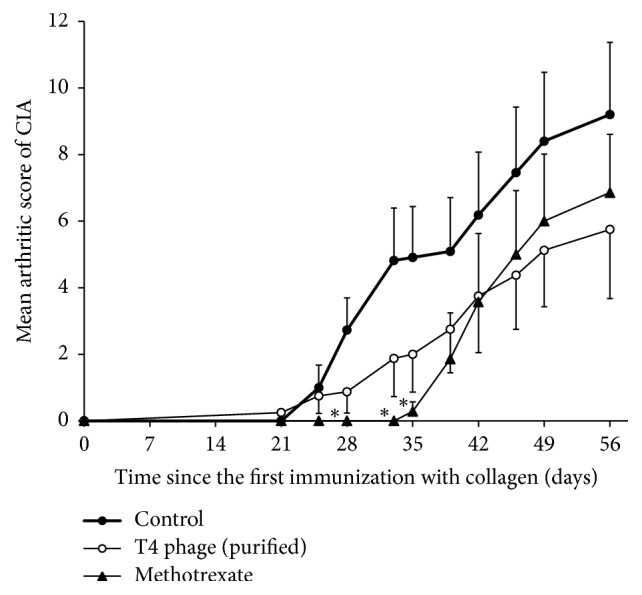
Influence of “therapeutic” intraperitoneal application of purified T4 phage preparation on the course of CIA. Dose of T4 phage in PBS: 5 × 10^8^ pfu/mouse once daily from day 22 until day 42 after the first dose of collagen. Control mice received 0.5 ml of PBS i.p. Number of mice in groups: *n* = 8–10. Methotrexate dose: 10 mg/kg three times a week (*n* = 7). ^*∗*^*p* < 0.05 in comparison to the control group (Mann–Whitney* U* test). For clarity reasons only upper or lower error bars representing SE are shown.

**Figure 5:**
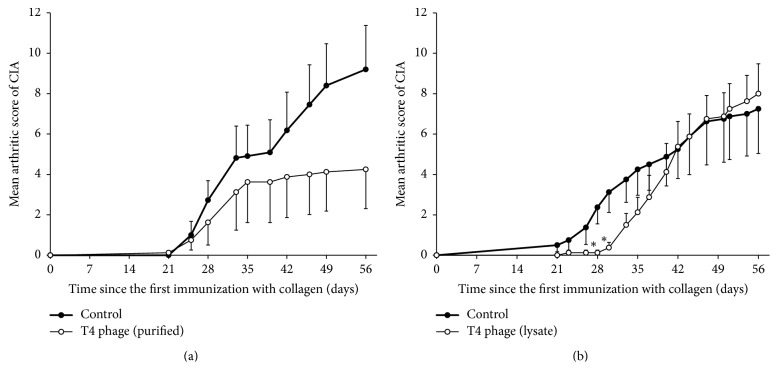
Influence of “prophylactic” intraperitoneal application (once daily from 3 days before until 5 days after the first dose of collagen) of purified T4 phage preparation and T4 phage lysate on the course of CIA. (a) Purified T4 phage in PBS: 5 × 10^8^ pfu/mouse (control mice received 0.5 ml of PBS i.p.); number of mice in groups: *n* = 8–10. (b) T4 phage lysate: 1 × 10^10^ pfu/mouse; number of mice in groups: *n* = 8. ^*∗*^*p* < 0.05 in comparison to the control group (Mann–Whitney* U* test). For clarity reasons only upper or lower error bars representing SE are shown.

We were also able to test the influence of purified T4 phage preparation obtained using the same technique as in our previously published studies on the course of arthritis (Figure 4) [8]. Its therapeutic application did not result in such marked inhibition of arthritis in mice as methotrexate, which was used as a positive control in this test. However, after day 56, we were able to observe a 37.5% CIA decrease in the T4 treated group, and it was significant when compared to the control group using the nonparametric Wald-Wolfowitz runs test. When the same phage was applied in prophylactic mode (from day –2 until day 5) the CIA inhibition was even lower (53.8%), but it did not show statistical significance (Figure 5(a)). However, T4 phage lysate application in this mode resulted in significant inhibition of CIA induction during the first two weeks after the booster dose of collagen (Figure 5(b)).

Additionally we performed an analysis of the influence of the phage lysate on weight, cell number, and lymphocyte populations of the thymus, spleen, and mesenteric lymph nodes 21 days after termination of its therapeutic application for the MS-1 staphylococcal phage mixture (Tables 2 and 3). We did not observe for MS-1 cocktail any significant differences compared to the control in the analyzed parameters of these organs (total body weight, weight of the organ, weight factor of the organ, and total cell number) besides a 29.4% increase in mean total number of splenocytes (Table 3). Application of the cocktail in mice also caused a 39% increase in the mean number of CD4-positive splenocytes when compared to the control (Table 3). However, this was also observed for a sonicate of the mixture of the bacterial host used for propagation of the phages, which are components of MS-1.

## 4. Discussion

The results of the therapeutic application of the phage lysates in the murine CIA model confirm the safety of the therapeutic bacteriophage application reported by human studies [1]. It should be underlined that phages were applied systemically at very high doses. The phage titer in the lysates used in this study was the highest one we could obtain during the standard process of production of these therapeutic phage preparations, which is usually between 10^6^ and 10^9^ pfu/ml [1]. The daily volume per kilogram of body weight of the preparation applied in an animal exceeded more than ten times the standard volume used for oral treatment according to our therapeutic protocol in humans, and the application lasted for three weeks. The staphylococcal phage lysates administered systemically (e.g., Staphage Lysate, also called SPL) are known to induce an immunological response in mammals, and bacterial lipopolysaccharides may potentiate CIA in mice after oral or systemic administration [6, 15, 16]. The minor increase in splenocyte number and percentage of their CD4-positive population three weeks after termination of application of staphylococcal phage lysate cocktail may reflect a well-known phenomenon of immunization with bacteriophages and formation of antiphage antibodies [17]. However, our results do not confirm that application of the phage lysates of Gram-positive (A3/R, MS-1,15/P) or high-load lipopolysaccharide containing Gram-negative (T4, 119x) bacteria could increase the process of immunization of mice with type II collagen and worsen the course of autoimmune joint disease. Moreover, prophylactic application of both Gram-positive (phi200) and Gram-negative (119x, T4) phage lysate weakened the process of immunization of mice with collagen and decreased the intensity of arthritis. As shown by the control experiment (Figure 3(c)), this unexpected immunosuppressive effect was not attributed to the phages themselves but rather to some of the components of broth culture used during the process of bacteriophage production.

Interestingly, the systemic therapeutic administration of the sonicate of the Gram-negative* P. aeruginosa* strain increased the CIA severity, whereas its lysate by 119x phage did not influence it significantly. This may result from the direct immunomodulatory effect of phages which we observed in an experiment when purified T4 phage was applied therapeutically. These observations are in accordance with our previous studies which showed that T4 phages were also able to mediate some anti-inflammatory activity not related to their ability to lyse bacterial cells such as inhibition of the production of reactive oxygen species by both endotoxin and live bacteria activated neutrophils, inhibition of the NF-*κ*B activity in herpes virus type-1 stimulated epithelial cells, and diminution of cellular infiltration of allogeneic skin allografts [8, 18]. Some of these effects may be attributed directly to the action of the phage proteins, as it has been recently described by Miernikiewicz et al. for tail adhesion gp12 of T4 phage [19].

## 5. Conclusion

Our preliminary results do not suggest that phages may aggravate the symptoms of rheumatoid arthritis. In contrast T4 phage may even exert an immunosuppressive effect in its mouse model.

## Figures and Tables

**Table 1:** Criteria of evaluation of inflammatory process in CIA.

Total points for a single paw	Corresponding arthritic score (degree of CIA severity in a single paw)
*0*	*0*
*1–3*	*1*
*4-5*	*2*
*6-7*	*3*
*≥8*	*4*

*CIA evaluation criteria and granted points:* (1) edema of any interphalangeal or metacarpophalangeal joint in a single toe—*1 point* for each involved toe; (2) moderate edema or redness of the metacarpal/metatarsus—*2 points;* (3) intensive edema of the metacarpal/metatarsus and wrist/ankle—*4 points;* (4) joint distortion *(ankylosis)*—*10 points.*

**Table 2:** Influence of MS-1 staphylococcal phage mixture on weight and cell number of thymus, spleen, and mesenteric lymph nodes 21 days after ending of its therapeutic application in CIA model in mice.

Parameter	Control			Bacterial sonicate			MS-1 (lysate)		
	*n*	Mean	±SE	*n*	Mean	±SE	*n*	Mean	±SE
Total body weight (g)	10	15.1	±0.3	10	15.7	±0.5	10	14.6	±0.2
Weight of thymus (mg)	10	21.0	±1.3	10	22.4	±1.9	10	21.7	±1.2
Weight factor of thymus (×10^−3^)	10	1.4	±0.1	10	1.4	±0.1	10	1.5	±0.1
Number of thymocytes (×10^3^/mm^3^)	10	282.0	±19.0	10	294.0	±23.3	10	292.0	±22.5
Weight of spleen (mg)	10	82.3	±1.6	10	86.4	±4.1	10	74.8	±4.9
Weight factor of spleen (×10^−3^)	10	5.5	±0.1	10	5.5	±0.2	10	5.1	±0.3
Number of splenocytes (×10^3^/mm^3^)	10	592.0	±38.1	10	682.0	±45.0	10	776.0	±57.3^*∗*^
Weight of mesenteric lymph nodes (mg)	10	29.3	±1.9	10	33.7	±2.9	10	26.6	±1.1
Weight factor of mesenteric lymph nodes (×10^−3^)	10	1.9	±0.1	10	2.1	±0.1	10	1.8	±0.1
Number of mesenteric lymph node cells (×10^3^/mm^3^)	10	314.0	±22.3	10	268.0	±20.0	10	332.0	±19.6

^*∗*^ *p* < 0.05 when compared to control group (one-way ANOVA, post hoc Scheffe's test).

**Table 3:** Influence of MS-1 staphylococcal phage mixture on lymphocyte populations of thymus, spleen, and mesenteric lymph nodes 21 days after ending of its therapeutic application in CIA model in mice.

Cell population	Phenotype	Control			Bacterial sonicate			MS-1 (lysate)		
		*n*	Mean	±SE [%]	*n*	Mean	±SE [%]	*n*	Mean	±SE [%]
Thymocytes	CD8+	10	2.1	±0.1	10	1.6	±0.2	10	2.3	±0.3
	CD4+	10	12.2	±0.9	10	12.4	±0.7	10	13.0	±1.1
	CD4+CD8+	10	79.6	±1.5	10	75.0	±7.7	10	77.9	±1.5
	CD4−CD8−	10	6.1	±0.7	10	3.0	±0.9	10	6.8	±1.2^*∗∗*^

Splenocytes	CD3+	9	17.5	±0.5	10	26.7	±4.6	10	22.1	±1.1
	CD19+	9	75.2	±1.5	10	67.2	±5.3	10	71.4	±0.9
	CD8+	10	2.4	±0.2	10	3.5	±0.3^*∗*^	10	3.3	±0.3
	CD4+	10	7.7	±0.4	10	11.3	±0.4^*∗*^	10	10.7	±0.5^*∗*^
	CD4+CD8+	10	2.6	±0.6	10	1.6	±0.4	10	1.6	±0.3
	CD4−CD8−	10	87.3	±0.9	10	83.6	±0.6^*∗*^	10	84.7	±0.9

Mesenteric lymph node cells	CD3+	9	32.7	±1.1	10	35.5	±1.2	7	35.6	±1.7
	CD19+	9	58.4	±2.5	10	57.7	±2.0	7	58.1	±1.2
	CD8+	9	4.8	±0.5	9	4.8	±0.2	7	5.1	±0.3
	CD4+	9	18.7	±0.7	9	24.6	±1.0^*∗*^	7	23.3	±2.1
	CD4+CD8+	9	6.7	±2.9	9	3.2	±0.4	7	2.6	±0.8
	CD4−CD8−	9	69.8	±3.1	9	67.4	±1.1	7	69.0	±2.0

^*∗*^ *p* < 0.05 when compared to control group (one-way ANOVA, post hoc Scheffe's test); ^*∗∗*^*p* < 0.05 when compared to bacterial sonicate treated group (one-way ANOVA, post hoc Scheffe's test).

## References

[1] Międzybrodzki R., Borysowski J., Weber-Dąbrowska B. (2012). Clinical aspects of phage therapy. *Advances in Virus Research*.

[2] Kingwell K. (2015). Bacteriophage therapies re-enter clinical trials. *Nature Reviews Drug Discovery*.

[3] Sansom C. (2015). Phage therapy for severe infections tested in the first multicentre trial. *The Lancet. Infectious diseases*.

[4] Kutter E., Borysowski J., Międzybrodzki R., Borysowski J., Międzybrodzki R., Górski A. (2014). Clinical phage therapy. *Phage Therapy: Current Research and Applications*.

[5] Górski A., Międzybrodzki R., Borysowski J. (2012). Phage as a modulator of immune responses: practical implications for phage therapy. *Advances in Virus Research*.

[6] Dean J. H., Silva J. S., McCoy J. L. (1975). In vitro human reactivity to staphylococcal phage lysate. *The Journal of Immunology*.

[7] Myers L. K., Rosloniec E. F., Cremer M. A., Kang A. H. (1997). Minireview: collagen-induced arthritis, an animal model of autoimmunity. *Life Sciences*.

[8] Międzybrodzki R., Świtala-Jeleń K., Fortuna W. (2008). Bacteriophage preparation inhibition of reactive oxygen species generation by endotoxin-stimulated polymorphonuclear leukocytes. *Virus Research*.

[9] Boratynski J., Syper D., Weber-Dabrowska B., Łusiak-Szelachowska M., Poźniak G., Górski A. (2004). Preparation of endotoxin-free bacteriophages. *Cellular and Molecular Biology Letters*.

[10] Ślopek S., Durlakowa I., Weber-Dabrowska B., Kucharewicz-Krukowska A., Dąbrowski M., Bisikiewicz R. (1983). Results of bacteriophage treatment of suppurative bacterial infections. I. general evaluation of the results. *Archivum Immunologiae et Therapiae Experimentalis*.

[11] Adams M. H. (1959). *Bacteriophages*.

[12] Rosloniec E. F., Cremer M., Kang A., Myers L. K., Coligan J. E., Kruisbeek A. M., Margulies D. H., Shevach S. W. (1996). Collagen-induced arthritis. *Current Protocols in Immunology*.

[13] Wooley P. H. (1988). Collagen-induced arthritis in the mouse. *Methods in Enzymology*.

[14] Suszko A., Obmińska-Mrukowicz B. (2013). Influence of polysaccharide fractions isolated from Caltha palustris L. on the cellular immune response in collagen-induced arthritis (CIA) in mice. A comparison with methotrexate. *Journal of Ethnopharmacology*.

[15] Caccese R. G., Zimmerman J. L., Carlson R. P. (1992). Bacterial lipopolysaccharide potentiates type II collagen-induced arthritis in mice. *Mediators of Inflammation*.

[16] Yoshino S., Sasatomi E., Mori Y., Sagai M. (1999). Oral administration of lipopolysaccharide exacerbates collagen-induced arthritis in mice. *The Journal of Immunology*.

[17] Łusiak-Szelachowska M., Żaczek M., Weber- Dąbrowska B. (2014). Phage neutralization by sera of patients receiving phage therapy. *Viral Immunology*.

[18] Górski A., Kniotek M., Perkowska-Ptasińska A. (2006). Bacteriophages and transplantation tolerance. *Transplantation Proceedings*.

[19] Miernikiewicz P., Klopot A., Soluch R. (2016). T4 phage tail Adhesin Gp12 counteracts LPS-induced inflammation in vivo. *Frontiers in Microbiology*.

